# Increased cardiac index due to terbutaline treatment aggravates capillary-alveolar macromolecular leakage in oleic acid lung injury in dogs

**DOI:** 10.1186/cc8137

**Published:** 2009-10-21

**Authors:** Raphael Briot, Sam Bayat, Daniel Anglade, Jean-Louis Martiel, Francis Grimbert

**Affiliations:** 1 Laboratoire TIMC, Equipe PRETA, Unité Mixte de Recherche 5525 du Centre National de Recherche Scientifique, Université Joseph Fourier, Centre Hospitalier Universitaire, Grenoble, 38043 cedex 09, France

## Abstract

**Introduction:**

We assessed the in vivo effects of terbutaline, a beta2-agonist assumed to reduce microvascular permeability in acute lung injury.

**Methods:**

We used a recently developed broncho-alveolar lavage (BAL) technique to repeatedly measure (every 15 min. for 4 hours) the time-course of capillary-alveolar leakage of a macromolecule (fluorescein-labeled dextran) in 19 oleic acid (OA) lung injured dogs. BAL was performed in a closed lung sampling site, using a bronchoscope fitted with an inflatable cuff. Fluorescein-labeled Dextran (FITC-D70) was continuously infused and its concentration measured in plasma and BAL fluid. A two-compartment model (blood and alveoli) was used to calculate KAB, the transport rate coefficient of FITC-D70 from blood to alveoli. KAB was estimated every 15 minutes over 4 hours. Terbutaline intra-venous perfusion was started 90 min. after the onset of the injury and then continuously infused until the end of the experiment.

**Results:**

In the non-treated injured group, the capillary-alveolar leakage of FITC-D70 reached a peak within 30 minutes after the OA injury. Thereafter the FITC-D70 leakage decreased gradually until the end of the experiment. Terbutaline infusion, started 90 min after injury, interrupted the recovery with an aggravation in FITC-D70 leakage.

**Conclusions:**

As cardiac index increased with terbutaline infusion, we speculate that terbutaline recruits leaky capillaries and increases FITC-D70 leakage after OA injury. These findings suggest that therapies inducing an increase in cardiac output and a decrease in pulmonary vascular resistances have the potential to heighten the early increase in protein transport from plasma to alveoli within the acutely injured lung.

## Introduction

Acute lung injury (ALI) is a major syndrome in patients in the intensive care unit, and it has a high mortality rate. An increased capillary-alveolar permeability to plasma proteins is an early marker of the acute phase of lung injury andcontributes to the development of fibroproliferation [1]and lung fibrosis, which both contribute to a negative outcome [2]. Plasma proteins that flood the alveoli include pro-coagulant factors and initiate a local coagulation cascade [3]. A relation between the early alteration of capillary-alveolar permeability to proteins and the fibrotic process has been confirmed in clinical studies [4]. The finding that in acute respiratory distress syndrome (ARDS) patients, bronchoalveolar lavage (BAL) protein levels decreased over time only in survivors suggests the involvement of the amplification of inflammatory responses due to alveolar protein flooding [5]. If a high BAL protein concentration in patients with ARDS predicts a higher risk of late fibrosis, any therapy aimed at reducing plasma protein accumulation in the interstitium and alveoli is of potential benefit.

We recently developed a modified BAL technique to monitor the capillary-alveolar leakage of macromolecules over several hours [6]. This technique allows the assessment of therapies aimed at slowing plasma protein accumulation in alveoli.

Several studies suggest a potential role for β2-agonists in the treatment of ARDS. These agents have been shown to reduce pulmonary neutrophil sequestration and activation, enhance surfactant secretion and modulate the inflammatory and coagulation cascades [7,8]. β2-adrenergic agonists have shown ability to reduce lung endothelial injury [9]and they are well known for their ability to enhance the epithelial fluid reabsorption by stimulating the activity and the expression of the epithelial sodium channels [10]. However, the results of clinical studies on β2-agonists effects in ARDS are controversial. The Beta-Agonist Lung Injury Trial (BALTI) [11]showed that intravenous albuterol treatment reduces extravascular lung water in patients with ARDS; but the recent study "AlbuteroL for the Treatment of ALI" (ALTA) [12] failed to find a beneficial effect of aerosolized albuterol therapy in a large randomized, placebo-controlled trial. Also, several *in vivo *studies showed no beneficial effects of β2-agonist therapy, in terms of protein accumulation in injured lungs [13,14]. Hemodynamic effects of β2-agonists may interfere with other potentially beneficial effects and may explain some of these contradictory results in patients with lung injury [15].

The goal of the present study was therefore to evaluate the effects of terbutaline, a widely used β2-agonist, on the early increase in macromolecular permeability of the capillary-alveolar barrier following lung injury. We used a well-known *in vivo *dog model of oleic acid (OA) lung injury in which we assessed the overall effect of terbutaline, both on protein leakage through the capillary-alveolar barrier and on pulmonary hemodynamics.

## Materials and methods

### Animal preparation

The experiments were performed on 19 anesthetized dogs. The experiments were carried out in accordance with the applicable French and European Community regulations. Animals were intubated and mechanically ventilated using 2% halothane to maintain anesthesia. Tidal volume was 10 mL/kg and respiratory frequency was adjusted between 12 and 20 breathes/minute to keep end-tidal CO_2 _within normal range. Fraction of inspired oxygen (FiO_2_) was adjusted to keep hemoglobin oxygen saturation above 95%, as measured by pulse oxymetry (SpO_2_). Arterial blood gases were measured every hour following a 10-minute period of ventilation with an FiO_2 _of 100%.

In injury experiments, animals were equipped with a pulmonary artery catheter and a catheter in the femoral artery. The following parameters were recorded: systemic arterial blood pressure (ABP), pulmonary arterial blood pressure (PAP), pulmonary arterial occlusion pressure (PAOP), and cardiac output (CO). Pulmonary capillary pressure (Pcap) was estimated from the back-extrapolation of the transitory pressure drop (between 0.2 and 2 seconds) following the inflation of the balloon of the pulmonary arterial catheter, using a double-exponential fit [16]. As control animals were destined for other later experiments, they were not equipped with invasive catheters.

### Broncho-alveolar lavage procedure

This modified BAL technique has been extensively described in a previous publication [6]. Briefly, in order to perform repeated BAL in a closed lung segment, an inflatable balloon was adapted to the extremity of a bronchoscope. Six initial BAL cycles were performed sequentially, in order to obtain a rapid saturation of the lavaged lung segment. Thereafter, one BAL cycle was performed every 15 minutes for the next three hours.

### Sample processing

Prior to the procedure, a batch solution of 500 mL of fresh lavage fluid was prepared by adding ^125^I-albumin (Cis Bio International, Paris, France) to saline (NaCl: 0.9 g %) at a final activity of 5 μCi/L as a dilution indicator of the lavage fluid inside the lung.

Thirty minutes before BAL, a 6 mg/kg bolus of a fluorescein isothiocyanate-labeled dextran (FITC-D70; average molecular mass, 73,100 daltons; Sigma, St. Quentin Fallavier, France) was injected, followed by an infusion of 6 mg/kg/hour in order to obtain a steady FITC-D70 concentration in plasma. Indicator concentrations were measured after the experiment. ^125^I-albumin activity was measured in BAL. FITC-D70 concentrations were measured in plasma and BAL fluid by fluorescence spectrophotometry, using excitation and emission wavelengths of 482 and 521 nm, respectively. The data analysis was performed using a two-compartment model where the FITC-D70 transport rate coefficient *K*_*AB *_(min^-1^) from blood to alveoli was estimated (see details of calculations in Additional data file 1).

### Experimental protocol

The study was performed in four separate groups.

In group 1 (n = 3) control animals were not injured with OA and received no terbutaline. In group 2 (n = 3) animals were not injured but received a terbutaline treatment.

In groups 3 and 4 the ALI was induced 30 minutes after the initial saturation BAL procedure. Every two minutes, 0.3 ml boluses of OA were injected into the superior vena cava through the proximal lumen of the pulmonary artery catheter up to a total dose of 0.08 ml/kg. Group 3 (n = 7) received OA injury without any treatment. In group 4 (n = 6) the terbutaline treatment was administered using an infusion of 0.25 mg/kg/min started 90 minutes after OA injury. Thereafter the terbutaline was continuously infused until the end of the experiment.

### Statistics

A statistical analysis was performed through Statview software (SAS Institute Inc. Cary, NC, USA). Group data are summarized as the mean ± standard error of the mean (SEM). First we compared global data by analysis of variance (ANOVA). Thereafter intra-group data were compared using a *post-hoc *test of Tukey-Kramer. Mann-Whitney rank-sum tests were used for two-group comparisons. We used linear regression models to determine individual correlations between *K*_*AB *_and hemodynamic data. Differences with a *P *< 0.05 were considered as significant.

## Results

The volume of the lavaged lung segment (***Vtn***) remained stable (63.1 ± 3.1 mL) throughout the sequential lavage cycles. The FITC-D70 plasma concentration was also stable throughout the experiment (0.15 ± 0.01 mg/mL).

**Figure 1:**
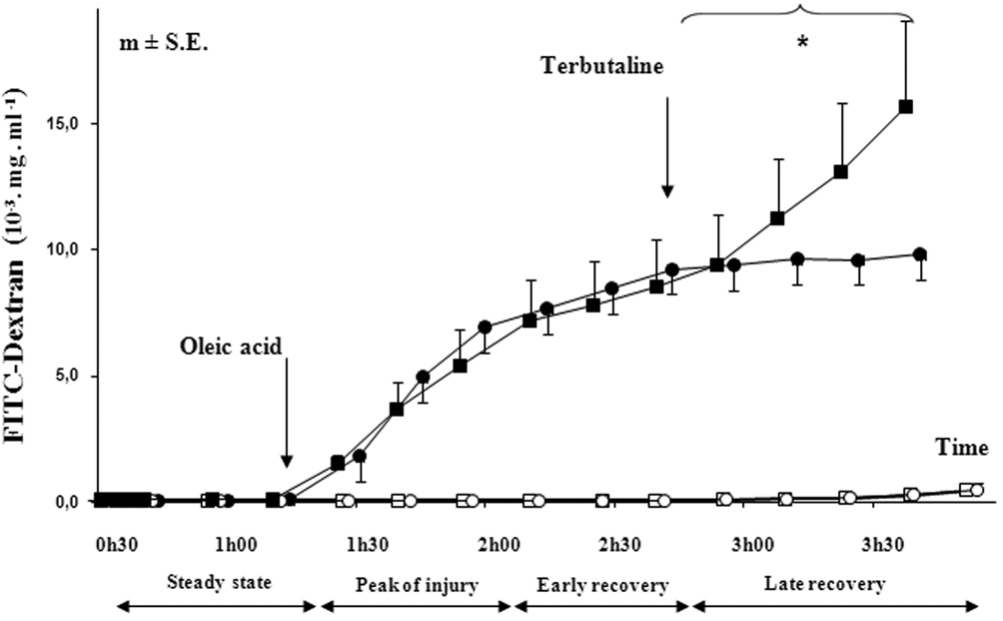
FITC-D70 concentration in broncho-alveolar lavage. Open circles = group 1 which was a control group (n = 3); Open squares = group 2 which was a control group with terbutaline infusion (n = 3); Filled circles = group 3 which was the OA injury group (n = 7); Filled squares = group 4 which was the OA injury with late terbutaline infusion group (n = 6). * *P *< 0.05 early recovery *versus *late recovery in group 4 (OA + terbutaline) (intra-group comparison by analysis of variance and the post-hoc test of Tukey-Kramer). FITC-D70 = fluorescein-labeled dextran; OA = oleic acid.

The alveolar concentration of FITC-D70 remained near zero in all groups before the onset of the injury. In injured animals (groups 3 and 4), the alveolar FITC-D70 concentration rose immediately after the OA infusion. In group 3 (OA) the FITC-D70 concentration reached a plateau during the last hour of the experiment, whereas in group 4 (OA + terbutaline) the start of terbutaline perfusion was followed by a second rise in BAL FITC-D70 concentration (Figure 1).

### Coefficient of capillary-alveolar leakage (*K*_*AB*_)

The FITC-D70 capillary-alveolar transport rate coefficient from blood to alveoli (*K*_*AB*_) was near zero in groups 3 and 4 before OA injury, a period defined as steady state.

Although *K*_*AB *_remained near zero in control groups 1 and 2 throughout the experiment, this coefficient rose markedly in OA-injured animals (groups 3 and 4) immediately after the onset of injury. *K*_*AB *_reached a peak value (peak of injury) 30 minutes after the OA infusion and decreased gradually thereafter. Within this recovery period, we distinguished two phases: early recovery (first 45 minutes after the peak of injury) and late recovery (last hour of the experiment).

**Figure 2:**
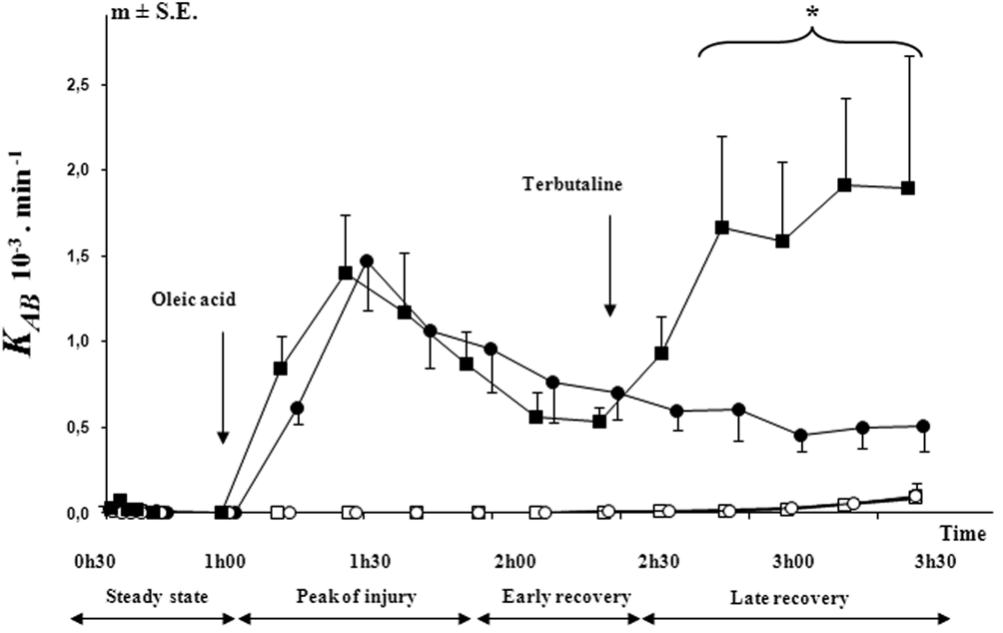
Time course of *K*_*AB*_, the transport rate constant for FITC-D70 from blood to alveoli. Open circles = group 1 which was a control group (n = 3); Open squares = group 2 which was a control group with terbutaline infusion (n = 3); Filled circles = group 3 which was the OA injury group (n = 7); Filled squares = group 4 which was the OA injury with late terbutaline infusion group (n = 6). _*_P < 0.05 early recovery *versus *late recovery in group 4 (OA + terbutaline) (intra-group comparison by analysis of variance and the post-hoc test of Tukey-Kramer). FITC-D70 = fluorescein-labeled dextran; *K*_*AB *_= coefficient of capillary-alveolar leakage; OA = oleic acid.

In group 3 (OA injury) *K*_*AB *_recovered slowly, while in group 4 (OA + terbutaline) *K*_*AB *_rose again after the onset of terbutaline administration (Figure 2).

### Hemodynamics values

In group 4 (OA + terbutaline) the cardiac index and the PAP increased and remained elevated following terbutaline administration. Such an increase was not observed in group 3 (OA). Total pulmonary vascular resistances (PVR) increased after OA injury and remained elevated in non-treated animals (group 3). Terbutaline infusion reduced the elevated PVR approximately to the pre-injury level. The hematocrit was constant throughout the experiment (mean value, 0.36 ± 0.1) with no significant difference between the different groups. The partial pressure of arterial oxygen (PaO_2_)/FiO_2 _ratio decreased markedly at the onset of the injury and did not recover later. There was no significant difference between groups at any stages of the experiment.

Hemodynamic and gas exchange data of groups 3 and 4 are summarized in Table 1.

**Table 1:** Hemodynamic and gas exchange data in groups 3 and 4

Mean ± SEM	Steady state 30 - 75 min	Peak 75 - 120 min	Early recovery 120 - 180 min	Late recovery 180 - 240 min
**ABP **(mmHg)				
Group **3**	95.4 ± 4.3	75.3 ± 9.0	84.7 ± 7.8	102.8 ± 3.3
Group **4**	93.0 ± 8.1	82.5 ± 4.6	93.8 ± 8.3	91.3 ± 3.1
**Cardiac index **(L/min/kg)				
Group **3**	0.16 ± 0.01	0.09 ± 0.01	0.10 ± 0.01	0.12 ± 0.01
Group **4**	0.14 ± 0.01	0.13 ± 0.01	0.13 ± 0.09	0.24 ± 0.02 *
**PAP **(mmHg)				
Group **3**	17.1 ± 1.2	13.2 ± 1.3	15.2 ± 1.3	18.5 ± 1.8
Group **4**	17.9 ± 1.9	18.4 ± 1.4	18.2 ± 1.5	24.2 ± 0.9 *
**Pcap **(mmHg)				
Group **3**	10.5 ± 1.5	10.5 ± 1.5	9.7 ± 1.1	10.3 ± 0.5
Group **4**	12.6 ± 1.6	14.0 ± 1.1	13.2 ± 1.3	15.6 ± 1.2
**PAOP **(mmHg)				
Group **3**	6.9 ± 0.6	6.3 ± 0.9	6.0 ± 1.0	6.0 ± 0.4
Group **4**	9.4 ± 1.5	9.3 ± 1.0	9.0 ± 1.7	10.0 ± 0.7
**PVR **(mmHg/L/min/kg)				
Group **3**	63.7 ± 7.6	76.8 ± 5.5	92.0 ± 10.2	104.1 ± 12.6
Group **4**	60.7 ± 11.5	70.1 ± 15.1	71.3 ± 12.1	58.9 ± 11.5 *
**PaO2/FiO2 **(kPa)				
Group **3**	36.2 ± 0.7	14.5 ± 4.4	10.5 ± 2.9	10.8 ± 3.7
Group **4**	27.9 ± 2.0	16.6 ± 2.7	15.3 ± 3.2	15.2 ± 3.1

ABP = systemic arterial blood pressure; FiO2 = fraction of inspired oxygen; PaO2 = partial pressure of oxygen in arterial blood; PAOP = pulmonary arterial occlusion pressure; PAP = pulmonary arterial blood pressure; Pcap = pulmonary capillary pressure; PVR = total pulmonary vascular resistances; SEM = standard error of the mean.

Group **3 **= OA injury without terbutaline (n = 7); Group **4 **= OA + terbutaline (n = 6); * *P *< 0.05 early recovery *versus *late recovery in group 4 (OA + terbutaline) (intra-group comparison by analysis of variance and the *post-hoc *test of Tukey-Kramer).

## Discussion

The main finding of this study is that the capillary-alveolar transport of FITC-D70 is increased by terbutaline infusion starting 90 minutes after the onset of an OA-induced lung injury. The participation of a terbutaline-induced increase in CO and PAP is suspected in the aggravation of lung injury.

### Methodological considerations

Our technique of capillary-alveolar permeability measurement modified a BAL technique previously described [17] and now allow the permeability to be monitored over extended periods of time. This new method has been fully described in a prior publication [6] and will be only briefly discussed here. This technique allows sampling of a lung segment saturated with BAL fluid every 15 minutes. Our BAL sampling technique offers a higher frequency of measurements and a greater sensitivity compared with the other techniques of lung permeability measurement either by lung lymph collection or the external radio-detection. This method differs also from the measurement of alveolar fluid clearance, which reflects the alveolar epithelium function, but variations are not necessarily correlated with the permeability to proteins of the capillary-alveolar barrier [18]. *K*_*AB*_, our transport rate coefficientof FITC-D70 from blood to alveoli, reflects the sum of capillary endothelial, interstitial and alveolar epithelial permeabilities arranged as resistances in series. It reflects also the perfusion surface area of lung capillaries. In normal conditions, thistransport rate coefficientis near zero. In this study, the time course and the values of *K*_*AB *_after OA injury were consistent with our prior findings [6] with a peak of injury followed by a slow recovery period. In this new series we studied specifically the effects of a terbutaline infusion started 90 minutes after OA injury. The terbutaline administration aggravated the capillary-alveolar transport of FITC-D70.

### Possible explanations for the observed terbutaline effects

The capillary-interstitial macromolecular flow through the endothelial barrier is essentially convective (i.e., drawn by capillary fluid filtration) when the endothelium is injured [19]. This elevation in convective transport can result from a decrease in the reflection coefficient for proteins of the capillary-alveolar barrier, or from an increase in capillary fluid filtration.

It is unlikely that the terbutaline diminished the reflection coefficient for proteins of the membrane. Indeed β-agonists are known for their anti-inflammatory effects and for improving the tightness of the endothelial cells [7,8].

**Figure 3:**
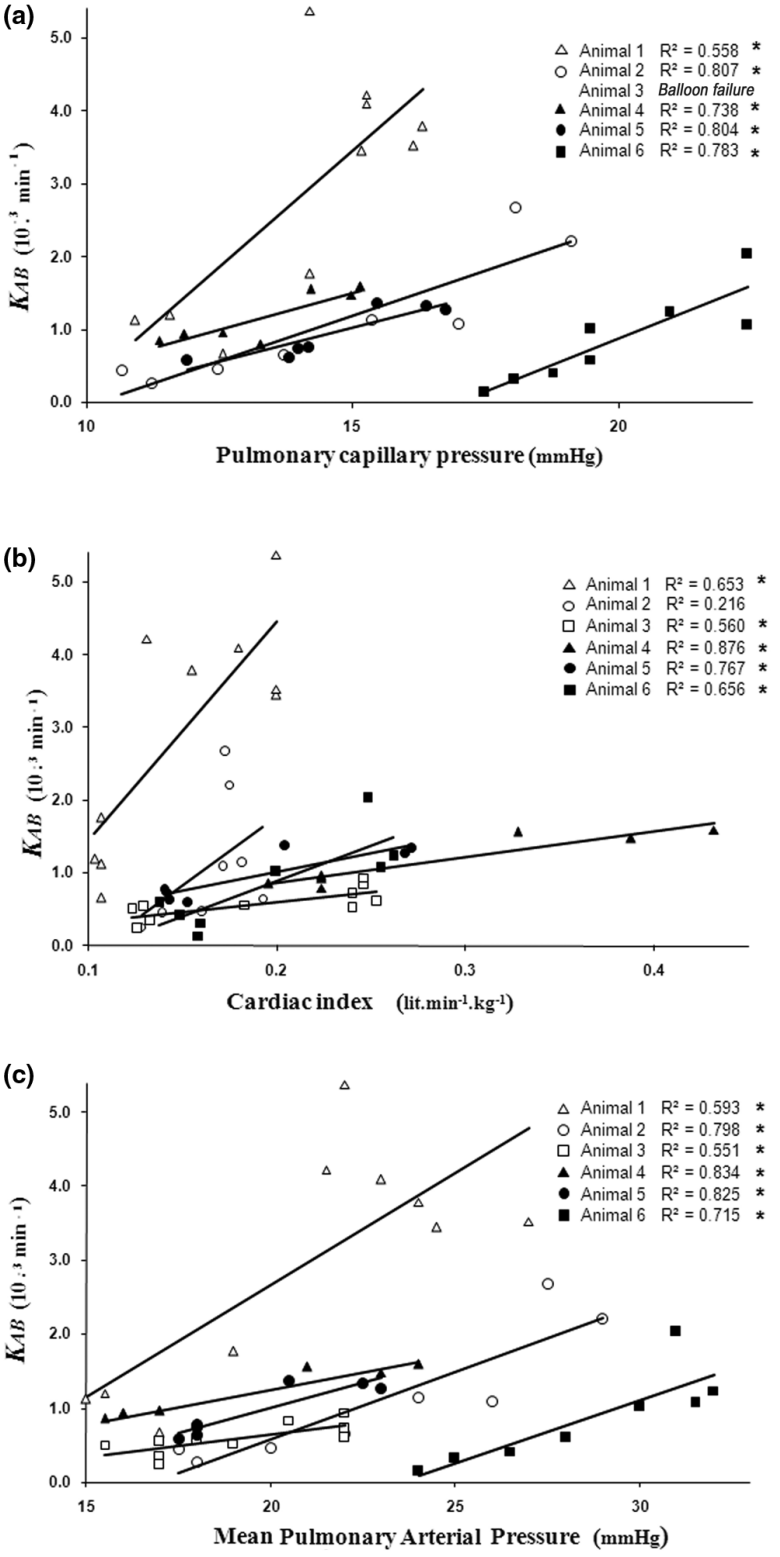
Correlations of *K*_*AB *_in group 4 (OA + terbutaline). Individual correlations in group 4 (OA + terbutaline) **(a) **between the FITC-D70 leakage index *K*_*AB *_and capillary pressure; **(b) **between *K*_*AB *_and cardiac index; and **(c) **between *K*_*AB *_and mean Pulmonary Arterial Pressure (by linear regression). FITC-D70 = fluorescein-labeled dextran; *K*_*AB *_= coefficient of capillary-alveolar leakage; OA = oleic acid.

Although β2-adrenergic agonists, such as terbutaline, stimulate water clearance by epithelial cells [10], we rule out a significant participation of fluid reabsorption in the post terbutaline rebound of FITC-D70 transport. Our BAL sampling process is designed to ensure a high alveolar fluid renewal (48%/h; see calculations in the Additional data file 1), largely higher than the potential alveolar epithelial fluid reabsorption, even when enhanced by terbutaline. On the other hand, terbutaline may have increased the fluid filtration through an elevation of the capillary pressure or an augmentation of the perfusion surface area. Indeed, our data have shown significant correlations in group 4 (OA + terbutaline) between the coefficient of FITC-D70 leakage *K*_*AB *_and Pcap, cardiac index and PAP (Figure 3).

We observed a non-significant trend towards an increased Pcap after terbutaline administration as compared with the early recovery period. In normal lung, an increase in Pcap leads to a large elevation in transvascular fluid filtration but a minor elevation in transvascular protein filtration [20]. In contrast, in lung injury entailing altered capillary-alveolar permeability, any increase in Pcap induces a large elevation in both transvascular fluid and protein filtration [21].

Terbutaline may also have increased the perfusion surface area and recruited leaky injured capillaries, which were initially derecruited by the hypoxic vasoconstriction. Several arguments plead in favor of this hypothesis. After terbutaline administration we observed a marked increase in cardiac index which is a well-known effect of the β-agonists. In normal lung, an increase in CO does not induce, by itself, an increase in transvascular fluid and protein filtration [22]. In contrast, in an OA-injured lung, increasing CO may worsen lung water accumulation likely by pulmonary vascular recruitment [23]. We also observed a significant reduction in PVR after terbutaline administration. Indeed, while OA injury initially elevated PVR, terbutaline administration reduced these elevated resistances to pre-injury levels. This drop in PVR, together with the increase in cardiac index, is consistent with an increase in perfusion surface area related to terbutaline infusion. b2-agonists are pulmonary vasodilators and terbutaline may also have lifted the hypoxic vasoconstriction and increased blood flow in injured lung zones, resulting in increased protein transport.

Therefore, we speculate that the rebound in FITC-D70 leakage, observed in our series during terbutaline perfusion, was provoked by an increase in perfusion surface area, associated to a small elevation of the Pcap.

### Recruitment of 'blind capillaries'

**Figure 4:**
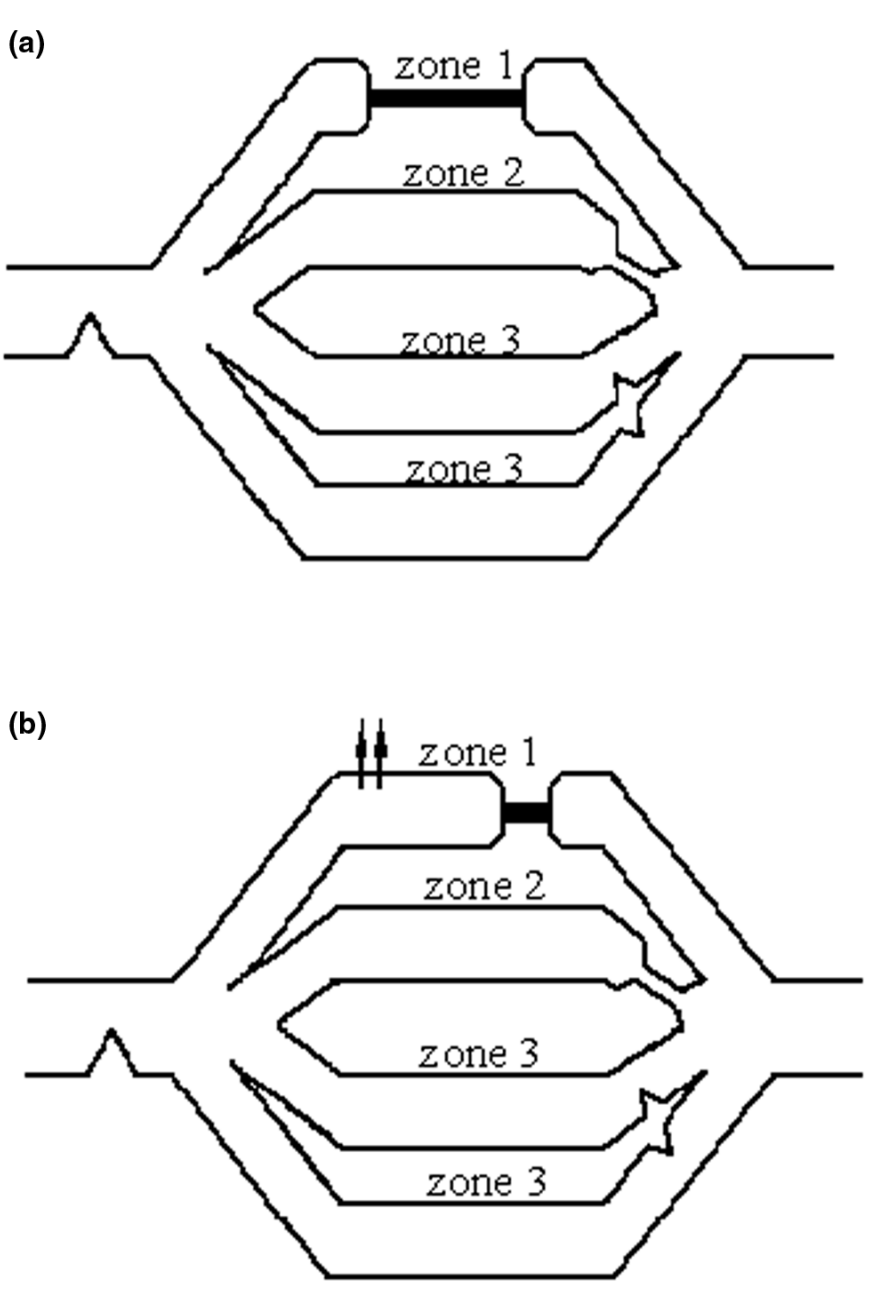
Recruitment of blind capillaries. Adapted from Anglade and colleagues [32] with permission. Model of circulation includes four branches of pulmonary circulation lying in parallel in same horizontal plane. Flow-limiting compression point in zone 2 lung is indicated by narrowing of vascular branch. **(a) **OA-injured lung before the terbutaline-increase in cardiac index and PAP. **(b) **Filling of blind vessels in zone 1, (i.e., opened at their arterial end and obstructed at their venous end). Corresponding injured capillaries may filter considerably (arrows) because filtration pressure in these non-flowing capillaries is at the level of pulmonary arteriolar pressure. In addition steep pressure gradient observed during increased cardiac index may also move downstream the obstruction point in zone 2 lung and increase filtration surface area. OA = oleic acid; PAP = pulmonary arterial blood pressure.

OA injury induces a hypoxic and mediator-induced active vasoconstriction, a perivascular compression by edema, and an intravascular obstruction by thromboembolism or endothelial swelling [24,25]. The active vasoconstriction is a pre-capillary and protective phenomenon. It accounts for approximately 50% of the pulmonary hypertension and is partially reversible. Eliminating this adaptive vasoconstriction response may dramatically deteriorate OA-injured lungs [26,27]. Several studies in intact dogs [28,29] have shown an increase in pulmonary venous resistance following OA injury. In the present study we also observed a trend, although not significant, towards an increase in pulmonary venous resistance during the peak of OA injury. This enhancement in postcapillary pulmonary venous resistance after OA injury may be mediated by thromboxane A2 release [30], sympathetic vasoconstriction [29], or microvascular obstruction [31]. Anglade and colleagues [32] have shown in OA-injured rabbit lung preparations that the increase in filtration surface area and capillary recruitment is larger when entailed by an elevation in CO than by an elevation in pulmonary venous pressure. These authors hypothesized that an elevation in CO could result in the re-opening of non-flowing leaky capillaries in zone 1, called 'blind capillaries' (i.e. opened at their arterial end and obstructed at their venous end) with a filtration pressure at the level of arteriolar pressure (Figure 4). This hypothesis is consistent with the observations of positron emission tomography (PET) imaging in OA injury, showing a greater reduction in blood flow in the most injured areas [33]. The injured capillaries are highly permeable vessels and their recruitment may considerably increase fluid and protein leak upstream of the obstruction.

In the present study, we speculate that the rebound of capillary-alveolar leakage, observed after terbutaline infusion, is consistent with the hypothesis of an arterial re-opening of blind injured capillaries in zone 1. Also, the elevation in CO and in PAP may have shifted downstream the obstruction point under zone 2 conditions towards the venous ends of capillaries and venules (Figure 4).

### Limitations of the study

Our preparation was designed only to study the early phase of the lung injury. BAL is known to cause a depletion in alveolar surfactant [34] and increase the recruitment of neutrophils [35,36], but it does not cause a significant change in the protein permeability of the epithelial barrier during the first four hours [37]. In the same manner, in our data, we observed a spontaneous aggravation of lung permeability, in control animals, when the experiments were prolonged beyond four hours [6]. Therefore, the BAL technique cannot be extended over a long period. Due to this time limitation, we cannot exclude that the rebound of FITC-D70 leakage, observed with terbutaline infusion, might be only a transient side effect of terbutaline, lately counterbalanced by β-agonist beneficial effects. Other studies have shown that β2-agonist action on capillary-alveolar membrane may be delayed for several hours. McAuley and colleagues [9] have observed a positive effect of salmeterol four hours, but not two hours, after the onset of an hydrochloric acid (Hcl) lung injury in rats. In the two recent large clinical studies on β2-agonist treatment in ARDS (BALTI [11] and ALTA [12]), patients were recruited approximately 24 hours after the beginning of lung injury and in the BALTI study, intravenous infusion of salbutamol reduces extravascular lung water in patients after a 72-hour delay.

Such delayed action might explain the lack of efficacy of terbutaline on capillary-alveolar permeability in our series. Other studies should be specially designed to evaluate the terbutaline effects beyond the first four hours following OA injury.

The OA injury model is also, by itself, a limitation of our study. OA is known to provoke a severe injury that probably overwhelmed the beneficial effects of β-agonists on capillary-alveolar permeability or edema fluid reabsorption. An aggravation of lung injury after an elevation in pulmonary blood flow has been frequently described in direct injuries of the lung such as OA injury [15,23,28,32]. In contrast, indirect lung injuries secondary to endotoxin infusion or severe sepsis seem to be less sensitive to variations in pulmonary blood flow and can be improved by β2-agonist therapies [38-40].

A final limitation of our study is the intravenous mode of terbutaline administration which may have provoked more hemodynamic effects than other routes. Aerosolized administration has been proposed to limit the systemic effects of the β2-agonists [41]. Atabai and colleagues [42] have shown that physiologically effective alveolar concentrations of salbutamol (10^-6 ^M) may be delivered with conventional systems in patients with pulmonary edema. However, the recent ALTA study [12] failed to find a beneficial effect of aerosolized albuterol therapy in a large randomized, placebo-controlled trial.

### Clinical implications

For everyday practice, our animal model may suggest that therapies inducing an increase in CO and a decrease in PVR have the potential to heighten the early increase in protein transport from plasma to alveoli within the acutely injured lung. Moreover, the removal of proteins from alveolar space is much slower than alveolar fluid clearance [43]. This accumulation of plasma proteins may contribute to the development of fibroproliferation [1] and lung fibrosis, which contribute to a negative outcome [2]. Our data are consistent with the notion, recently emphasized by experimental [15] and clinical [44] publications, that the vascular side of the capillary-alveolar membrane cannot be ignored.

## Conclusions

Our BAL technique allowed the monitoring of the time course of capillary-alveolar barrier leakage of macromolecules and it's recovery in a pre-clinical model of ARDS. We found that an infusion of terbutaline, started 90 minutes after the onset of OA injury, increased capillary-alveolar transport of FITC-D70. We speculate that the hemodynamic effects of terbutaline, may have contributed to this increased leakage of macromolecules, and to the recruitment of leaky capillaries thereby increasing the capillary exchange surface area. These findings suggest that therapies inducing an increase in CO and a decrease in PVR have the potential to heighten the early increase in protein transport from plasma to alveoli within the acutely injured lung.

## Key messages

• Capillary-alveolar permeability to macromolecules (FITC-D70) was measured every 15 minutes for 3 hours, in an OAARDS model in anesthetized dogs.

• The time course of injury showed a peak of permeability 30 minutes after the OA infusion, followed by a slow recovery.

• A treatment by terbutaline intravenous infusion, started 90 minutes after the onset of OA injury, increased capillary-alveolar permeability to FITC-D70.

• Hemodynamic effects of terbutaline, may have recruited leaky capillaries and increased the capillary exchange surface area.

• Therapies inducing an increase in CO and a decrease in PVR may aggravate the protein leakage from plasma to alveoli within the acutely injured lung.

## Abbreviations

ABP: systemic arterial blood pressure; ALI: acute lung injury; ALTA: AlbuteroL for the Treatment of ALI; ANOVA: analysis of variance; ARDS: acute respiratory distress syndrome; BAL: broncho-alveolar lavage; BALTI: Beta-Agonist Lung Injury Trial; CO: cardiac output; FiO2: fraction of inspired oxygen; FITC-D70: fluorescein-labeled dextran; Hcl: Hydrochloric acid; *K*_*AB*_: coefficient of capillary-alveolar leakage; OA: oleic acid; PaO2: partial pressure of oxygen in arterial blood; PAOP: pulmonary arterial occlusion pressure; PAP: pulmonary arterial blood pressure; Pcap: pulmonary capillary pressure; PET: Positron emission tomography; PVR: total pulmonary vascular resistances; SEM: standard error of the mean; SpO2: pulsed oxygen saturation; *Vtn*: volume of the lavaged lung segment.

## Competing interests

All the authors of this paper declare that they have no competing interests.

## Authors' contributions

RB collected the samples and data, performed the data analysis, and wrote the initial draft and the final manuscript. SB and DA participated in data collection and in revising the final manuscript.

JLM directed the mathematical analysis and participated in drafting the initial manuscript. FG conceived the premise and participated in data collection, interpretation and analysis, and in revising the final manuscript. All authors have read and approved the final manuscript.

## Supplementary Material

Additional file 1 Word file with a proposed explanation of the two-compartment model used to interpret the data and detail the calculation of the capillary-alveolar leakage coefficient *K*_*BA*_. Click here for file
