# Supplementary material for: Increased cardiac index due to terbutaline treatment aggravates capillary-alveolar macromolecular leakage in oleic acid lung injury in dogs
Source: Crit Care. 2009 Oct 21;13(5):R166. doi: 10.1186/cc8137 (PMC2784397; doi:10.1186/cc8137)
Supplement: Additional file 1 — Word file with a proposed explanation of the two-compartment model used to interpret the data and detail the calculation of the capillary-alveolar leakage coefficient KBA. [file cc8137-S1.doc]

**Additional data file 1**

The lavaged lung segment volume (*V_t,n_*) at cycle *n* (*n,* lavage cycle number) is calculated from the alveolar ^125^I-albumin concentration :

$$V_{t,n}=\text{ }\frac{Q_{n}}{C_{r,n}}$$

where *C_r,n_* is the activity of ^125^I-albumin per ml of retrieved BAL fluid, *Q_n_*; the amount of ^125^I-albumin added to the lung segment (*Q_a,n_*) minus the amount removed due to sampling of the BAL fluid (*Q_s,n_*) :

In order to simplify the mathematical formalization of the two-compartment model (blood and alveoli), a constant blood FITC-D70 concentration is assumed. The mass exchanges of FITC-D70 between the blood and alveolar compartments depend on two transport rate coefficients. The first, named *K_AB_*, is the transport rate coefficient (min^-1^) for FITC-D70 from blood to alveoli; the second, named *K_BA_*, is the transport rate coefficient (min^-1^) for FITC-D70 from alveoli to blood.

Since the volume of the BAL fluid samples (*V_FS_*) was as large as 10% of the lavaged lung volume and was replaced by fresh lavage fluid, the FITC-D70 concentration gradient from blood to alveoli was maintained. The return FITC-D70 mass flow from alveoli to blood (dependent upon the transport rate coefficient *K_BA_* in the two-compartment model) was therefore considered negligible. The removal of FITC-D70 from the alveolar compartment due to the BAL fluid sampling process (including its replacement with fresh fluid) was calculated for each BAL cycle as dependent upon a fluid sampling transport rate coefficient (min^-1^), named (*K_FS_*), such as :

$$K_{FS}\text{ }=\text{ }\frac{V_{FS}}{V_{t,n}\text{ }.T}$$

where *T* is the cycle duration.

The mass balance of the system is given by :

*d* [*FITC-D70*]*_Blood_*

= 0

*dt*

*d* [*FITC-D70*]*_Alveoli_*

= *K_AB_ (t)* [*FITC-D70*]*_Blood_* – (*K_FS_ (t)* + *K_BA_ (t)* [*FITC-D70*]*_Alveoli_*)

*dt*

For each BAL cycle of rank *i*, we define a time interval, named *T_i_ = t_i+1_ - t_i_*, where *t_i+1_* is the beginning of the cycle *i+1*. We assume that *K_FS_* and *K_BA_* are constant within *T_i_*. In order to simplify the solution of the above system of differential equations, we introduce: *X(t) =* [*FITC-D70*]*_alveoli_* at time *t* and *Y(t) =* [*FITC-D70*]*_blood_*, that we assume constant during the experiment. The solution is :

where *K_FS,i_* is the removal transport rate coefficient of FITC-D70 from the alveolar compartment due to the BAL fluid sampling process during the time interval *T_i_* ; *K_AB,i_* is the transport rate coefficient for FITC-D70 from blood to alveoli during the time interval *T_i_*. These two coefficients are assumed to be constant during the time interval *T_i_*. The unknown *K_ABi_* is estimated from the above solution by :

*K_ABi_* is expressed as the transport rate coefficient (min^-1^) for FITC-D70 from blood to alveoli.
